# A farewell to Co-Editor-in-Chief (Nov. 14, 2025)

**DOI:** 10.1038/s41377-025-02129-w

**Published:** 2025-11-27

**Authors:** Xi-Cheng Zhang

**Affiliations:** The Institute of Optics, University of Rochester, Rochester, NY USA

**Keywords:** Applied optics, Optics and photonics

## Abstract

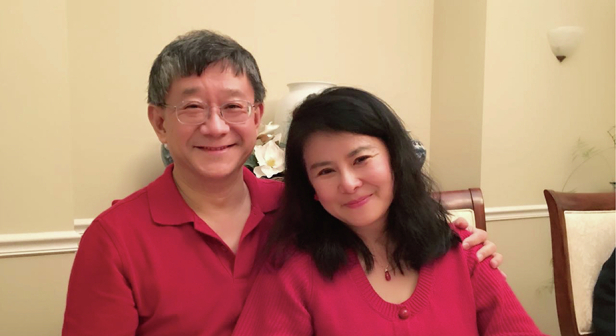

On 14 November 2025, I will formally step down as Co–Editor-in-Chief of *Light: Science & Applications*. This decision, reached after lengthy discussions with the administrators at the University of Rochester, our Research Security Officer and our Global Operations Director, aligns with my forthcoming institutional responsibilities.
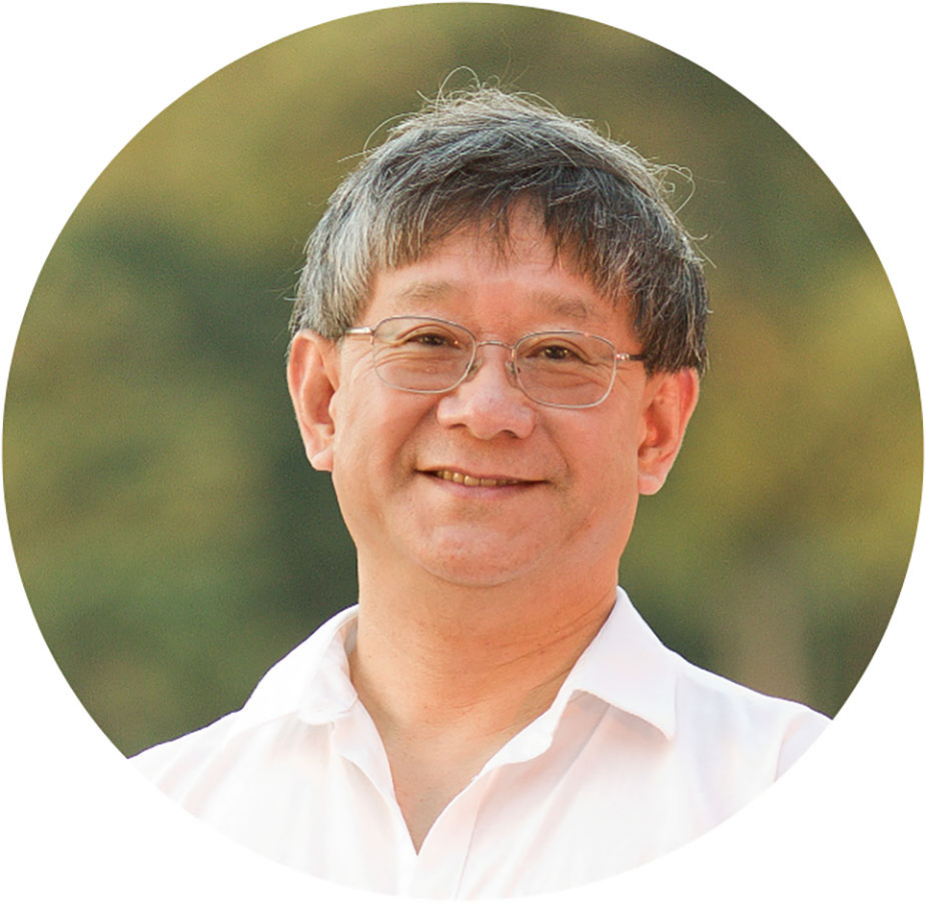


It has been a tremendous honour since 2020 to serve alongside an outstanding editorial team—first as Executive EiC (2020–2022) and subsequently as Co-EiC (2022-2025). I am deeply grateful to our founding Editor-in-Chief Jianlin Cao, to Executive Editor Yuhong Bai, to the full LSA Editorial Board, and the office staff at Springer Nature for enabling the journal to achieve new heights.

When I assumed the Executive EiC role in 2020, LSA had already reached a strong position in the optics/photonics field—thanks to Jianlin Cao’s leadership, the dedicated Board and staff, and the strong base of manuscript submissions, impact factor growth, and reputation. Even so, through our team’s efforts we were able to build on that momentum in several dimensions:

The journal’s Impact Factor rose from ~17.78 in 2020 to ~20.6 in 2023 and ~23.4 (2024). This continued upward trajectory reinforced LSA’s standing in the top tier (Q1) of optics journals. At the same time, the volume of manuscripts, the international reach of authors, and the global visibility of LSA continued to expand.

Together with the office staff, we implemented more frequent communications between the EiC team and office staff, strengthening the workflow and responsiveness of the editorial office. We refined our acceptance criteria—to ensure rigour without sacrificing speed—and clarified expectations for authors, reviewers and board members. We expanded the Editorial Board in both size and geographic diversity, inviting more rising and established experts in emerging sub-fields of optics and photonics. We introduced periodic editorial board meetings (virtual or hybrid) to foster stronger engagement with the board.

We launched several new Special Issues and thematic collections to capture emerging frontiers (for example in green photonics, integrated devices, quantum photonics and biophotonics). These special issues helped focus attention, recruit high-impact content and draw broad readership. We also strengthened our outreach via the “Light People” column (interviews with leading scientists/engineers), and correspondences/discussion pieces aimed at stimulating community dialogue.

Under our tenure we emphasized global engagement and author service: faster decision-turnaround, enhanced transparency of peer-review, and improved author metrics/visibility (e.g., via highlight articles/news & views). The editorial office travelled or participated in a greater number of international optics/photonics conferences, helped nurture younger authors and reviewers, and extended LSA’s network of reviewers and board members. As one article puts it, “the secret to the success of Light … is the mutual trust between our editorial staff and high-level researchers” and its wide distribution of editorial board members and global offices.

I have been honoured to receive the LSA 2025 Outstanding EiC Award—an acknowledgement not of personal achievement alone but of the collective success of the editorial team, board, office staff and authors.

My six years of editorial service at LSA have offered extraordinary opportunities: to engage with a global network of optics and photonics researchers; to witness frontier breakthroughs (from metamaterials to ultrafast optics to quantum photonics); to collaborate with a dedicated team of editors, board members and staff; and to attempt to keep precision, fairness and speed at the heart of every editorial decision.

I have found that strong journal leadership is not just about metrics but about relationships: authors trust the editorial office when they see fairness; reviewers engage when they feel their work is valued; board members contribute best when they feel connected to the mission. I am grateful to all of you—authors, reviewers, board members, office staff, and my Co-EiC colleagues—for your trust and partnership.

I look with confidence to LSA’s future under new leadership. I am fully confident that the journal will continue to flourish, adapt and lead in the fast-moving field of optics and photonics.

In closing, thank you to everyone who contributed to LSA’s journey—past, present and future. I would like to use this Farewell note to thank my wife Wendy Zhang, she has always been with me during my most difficult period, especially for my spinal surgery and recovery. Without Wendy’s unconditional love and support, I am not able to carry EiC role for sure.
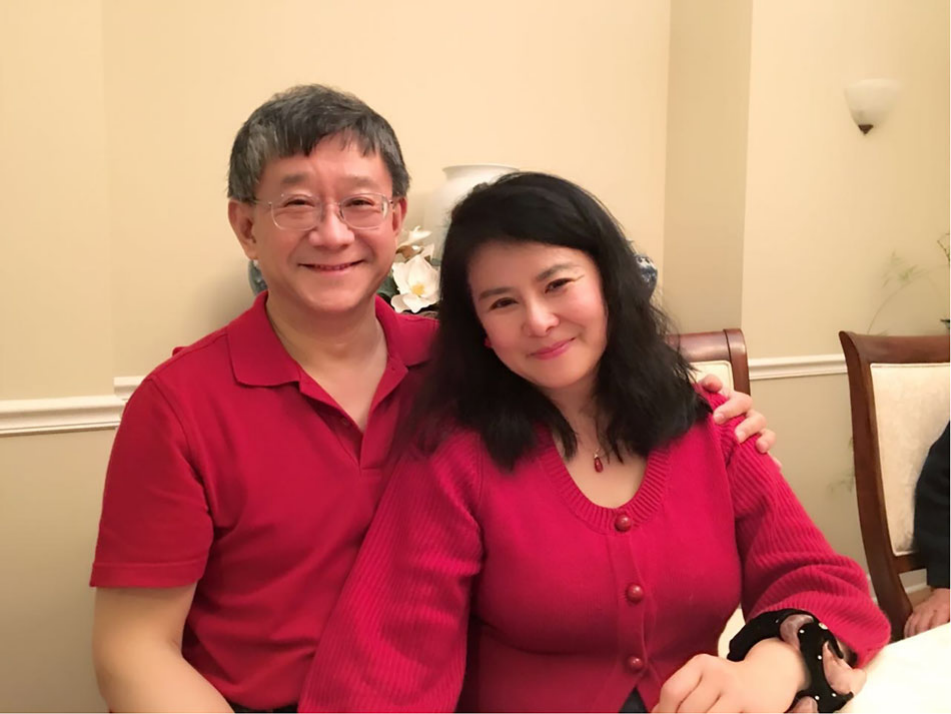


Xi-Cheng Zhang and his wife Wendy

I wish all the best to my co-EiC, Yun-Feng Xiao, and I look forward to following the journal’s continued success.

With warmest regards,

Xi-Cheng Zhang

Co-Editor-in-Chief, *Light: Science & Applications*

Institute of Optics, University of Rochester

